# Childbearing desires and behaviour: a prospective assessment in Nairobi slums

**DOI:** 10.1186/s12884-019-2245-3

**Published:** 2019-03-28

**Authors:** Kazuyo Machiyama, Joyce N. Mumah, Michael Mutua, John Cleland

**Affiliations:** 1 Faculty of Epidemiology and Population Health, London School of Hygiene and Tropical Medicine, Keppel Street, London, WC1E 7HT UK; 2 African Population and Health Research Center, APHRC Campus, 2nd Floor, Manga Close, Off Kirawa Road, P.O. Box 10787-00100, Nairobi, Kenya

**Keywords:** Sub-Saharan Africa, Kenya, Fertility preferences, Predictive validity

## Abstract

**Background:**

Advancing an understanding of childbearing desires is an important precursor to achievement of the policy goal of reducing unintended pregnancies. It has been long debated that concepts of fertility desires and planning may be particularly problematic in sub-Saharan Africa. However, examination of the utility of fertility preference measures and their link to reproductive behaviour is still rare in the region. The aim of this study is to assess the predictive validity of future childbearing desires on subsequent reproduction among women living in the highly unpredictable circumstances of Nairobi slums.

**Methods:**

We used data from a longitudinal study (2007–2010) nested in the Nairobi Urban Health Demographic Surveillance System that is located in two slums in Nairobi, Kenya. We analysed baseline fertility desires among 4577 postpartum women. Cox proportional hazard model was employed to examine the effect of fertility desires on subsequent reproduction.

**Results:**

One-third of the women wanted no more children and 37% wanted to wait for at least five years at baseline. While two-thirds of the women who wanted to have a child soon became pregnant within three years, less than one-third of those wanting no more children became pregnant. The multivariable analysis shows that the probability of becoming pregnant among women who expressed desires to stop or delay childbearing at least for five years was 50% less than among women who wanted to have a child in two to four years. In addition to prospective fertility desires, level of woman’s education, residence and ethnicity exerted important influences on implementation of baseline preferences.

**Conclusions:**

Our study finds a strong link between baseline fertility desires and subsequent reproduction. A large difference in pregnancy risk was observed between those who wanted no more children and those who wanted another child. The link between a woman’s stated desire to stop childbearing and subsequent childbearing is just as strong in the Nairobi slums as elsewhere. In addition, the findings revealed a pronounced gradient in pregnancy risk according to preferred spacing length, which support other evidence on the important contribution of long-term spacing or postponement to fertility decline in sub-Saharan Africa.

## Background

For decades, the concept of unmet need for contraception has provided the single strongest argument for investment in family planning in low- and middle-income countries. Though its precise measurement is complex, the central idea is straightforward: unmet need refers to women who state that they want no more children or wish to delay the next child for at least two years but are using no method of contraception. It has long been acknowledged that these simple questions on future childbearing preferences cannot capture nuance, ambivalence, emotional orientation and uncertainty of sentiments [1, 2].

It has been argued that the concepts of fertility preferences and planning may be particularly problematic in sub-Saharan Africa. Preferences may be ambivalent. For instance, among women in Burkina Faso, Ghana and Kenya who did not want a child at all, appreciable minorities of women reported that they would experience no problem if they became pregnant in a few weeks and about 10% of women wanting to delay the next childbearing said that they would be happy with an early pregnancy [3]. Preferences may also be unstable. A high degree of fluctuation in reproductive desires was reported among young women in longitudinal studies in Kenya, Ghana and Malawi [4–7]. Johnson-Hanks and Sennott and Yeatman argued that fertility preferences of young women in Cameroon and southern Malawi may be tentative and unstable as an adaptation to unpredictable environments [5, 8].

Unpredictability of life is likely to especially be high in slums. Slum dwellers often face pervasive poverty, congestion, inadequate services, personal violence and breakdown of relationships. In our study setting, Korogocho and Viwandani, two Nairobi slums, the majority of adults is engaged in casual work; in 2009 only 14% were engaged in salaried employment and one-quarter were economically inactive. In contrast to rural areas where many families rely on farming and grow much of their own food, the urban slum population relies on the market economy and needs to buy food in addition to paying rent and other costs. For example, a study by Macharia et al. found that only 20% of households are food secure in Korogocho and Viwandani [9]. Additionally, not only household incomes but also conjugal relationship are unstable in these settings and divorce and separations are more common in slums than elsewhere in Kenya [10].

In African cities, women experience a constant dilemma between concerns for the costs of an additional child and the desire for a larger family [11–13]. Having an additional child in these highly uncertain circumstances may put extra pressure not only on their household economic circumstances but also the spousal relationship [11]. Women who want to postpone childbearing for a long time or do not want any more children may also have conflicted preferences. They may revise their preferences if their partner or partner’s family want more children or if financial circumstances improve. These considerations imply that the link between desired future childbearing and subsequent behaviour may be weak among slum dwellers.

In spite of the aforementioned concerns, examination of the utility of fertility preference measures and their link to reproductive behaviour is still rare in Africa. While an increasing but small number of prospective studies assessed to what extent fertility preferences predict future reproductive outcomes among women in sub-Saharan Africa [14–17], none has been conducted in urban informal settlement settings.

Sub-Saharan Africa will account for one-half of world population increase between 2015 and 2050 [18]. The population growth will occur almost entirely in urban areas. It is projected that over the next three decades (2020–2050), Africa will be the fastest urbanising region in the world [19]. While many new arrivals in the outlying informal neighbourhoods of cities are migrants from rural areas, a large part of this growth will be due to the larger number of births than deaths among urban residents. Inequality in cities is higher than in rural areas, and most cities are falling behind in housing provision and improving vital infrastructure compared with the rapid pace of population growth. It is reported that a majority of the urban population (62%) in sub-Saharan Africa live in slums or in slum-like conditions [20]. While the urban fertility rate has fallen in recent years, urban unmet need for modern contraception and unintended pregnancy are still high. The rapid growth in the number of women of reproductive age living in cities with limited access to vital services such as family planning creates enormous need for targeted sexual and reproductive health services.

Advancing an understanding of childbearing desires is an important precursor to achievement of the policy goal of reducing unintended pregnancies. The aim of this paper, therefore, is to assess the extent to which baseline childbearing preferences predict subsequent reproductive outcomes among women aged 15–49 years in two slums in Nairobi, Kenya, using longitudinal data from the Nairobi Urban Health Demographic Surveillance System (NUHDSS). To our knowledge, this is the first prospective analysis to assess predictive power of fertility preferences in urban slums in sub-Saharan Africa.

## Methods

### Study setting

In Nairobi, an estimated 60–70% of the population live in informal settlements or slums [10]. This study focuses on two of the slums in Nairobi – Korogocho and Viwandani, where the African Population and Health Research Center (APHRC) has operated the NUHDSS since 2002. The NUHDSS observes approximately 66,000 people in about 27,000 households as of December 2012; a detailed description of the HDSS has been published elsewhere [21]. Viwandani represents a youthful and mobile population, with 95% of residents being migrants from rural areas and majority of residents seeking jobs in the nearby industrial area. Korogocho is the fourth largest slum in Nairobi and a more settled community. About one-quarter of the residents were born and raised in the settlement. Despite the high levels of mobility, the average durations of residence are 14 years in Korogocho and eight years in Viwandani [22]. As mentioned earlier only a minority has salaried employment and about one-half of adults relies on unstable sources of income with one-half of women is classified as economically inactive [22]. Some improvements have been observed in the health status of Nairobi slum dwellers, but indicators remain well below those of Nairobi overall. For instance, the mortality of children under five was 80 per 1000 live births according to the 2012 Nairobi Cross-sectional Slums Survey (NCSS), while the average in Nairobi according to the 2014 Demographic Health Survey (DHS) was 52 per 1000 live births [23].

According to the 2008 Kenya DHS, the total fertility rate in Nairobi was 2.8. Among currently married women aged 15–49, one-half was using a modern contraceptive method (49%); 48% wanted to stop childbearing and 26% wanted to delay the next child for two or more years [24]. Although Nairobi has low prevalence of unmet need for family planning (15%) compared with the other regions in the country, one-third of current and recent pregnancies was classified as unintended (17% unwanted and 14% mistimed).

The 2012 NCSS estimated the total fertility rate in the Nairobi slums to be 3.5 and modern contraceptive prevalence to be 54%, a little higher than the prevalence estimated for Nairobi City from the 2008 Kenya DHS [10]. The proportion of women in the slums who wanted to delay childbearing was higher than that in the whole city (39% wanted to stop childbearing and 35% wanted to delay). One-third of pregnancies were either mistimed or unwanted (10% unwanted and 24% mistimed) [10]. The data from the two surveys suggest that women in Nairobi slums enter marriage and start childbearing early, and one-third of women want to space childbearing but become pregnant sooner than they prefer despite the high contraceptive prevalence.

### Data

We used the data from the Maternal and Child Health (MCH) component of a broader research project called *Urbanization, Poverty and Health Dynamics* that was nested in the NUHDSS. Children born between September 2006 and October 2010 and their mothers or guardians who lived in the NUHDSS sites were eligible for enrolment in the study. Data collection started in February 2007 and follow-up interviews took place every four months until November 2010. Interviews were conducted in Swahili or English. Trained fieldworkers recruited new children at every visit during the study period, and new children together with their mothers or guardians formed new cohorts at every round of data collection. Thus, women who had additional births during the follow-up period were included in multiple cohorts. Because of a high rate of out-migration from the NUHDSS, death of the woman or the child, failure to find the woman and refusals, initial enrolment into the MCH study covered only 73% of all known births [25]. A relatively high level of attrition, mainly due to migration, was experienced in this study, ranging on average from 21 to 28% per year [26].

The MCH module covered maternal and child health and reproductive events, such as antenatal and postnatal care, feeding practice, anthropometric measures of mothers and children, month-to-month calendar data on contraceptive use, breastfeeding and postpartum sexual behaviours and amenorrhea.

The primary outcome of this study is pregnancy conceived between the baseline and the last observation in the MCH module. The MCH module ascertained the current pregnancy status and gestational age of every woman in the study site at every visit. Because of potential underreporting of pregnancies particularly in the first trimester [27], additional pregnancies were identified from the routine vital registration data in the NUHDSS. In these cases, we computed an approximate date of conception by subtracting 280 days from the child’s date of birth. Although use of both MCH and the vital registration data increased the coverage, there will inevitably be underreporting of pregnancies that were terminated or ended in miscarriage.

Women were asked about their future childbearing desires at the time when mother-child dyads were initially enrolled in the study. The precise question was “would you like to have another child or would you prefer not to have any more children?” A preferred timing of the next birth was elicited from women who said they wanted to have another child (“for how long would you like to wait before you have another child?”). The study also assessed the strength of fertility desires. At baseline, women were asked, “in the next few weeks, if you discovered that you were pregnant, would that be a big problem, a small problem or no problem for you?”. Women who did not want a child soon were also asked to provide the main reason for their desires.

The analyses were restricted to women aged 15–49 at baseline who were residents in the NUHDSS. Residency in the NUHDSS is defined as having lived in the surveillance locality continuously for 120 days. In this analysis, 25 sterilised women, 10 women who declared that they could not get pregnant, 23 women who were pregnant at baseline and eight women whose index child had died before the baseline interview were excluded.

### Analysis

Survival analyses were applied in order to estimate the cumulative probability of pregnancy according to prospective fertility desires. Hazard ratios of pregnancy were computed using Cox proportional hazards models to examine the effect of these fertility desires on the hazards of pregnancy. All analyses were adjusted for key socio-demographic background characteristics. We also repeated the analysis focusing on women who wanted to wait for five or more years and those who wanted no more children to assess the predictors of implementation of these preferences. Stata 14.0 was used for the analysis.

## Results

**Fig. 1:**
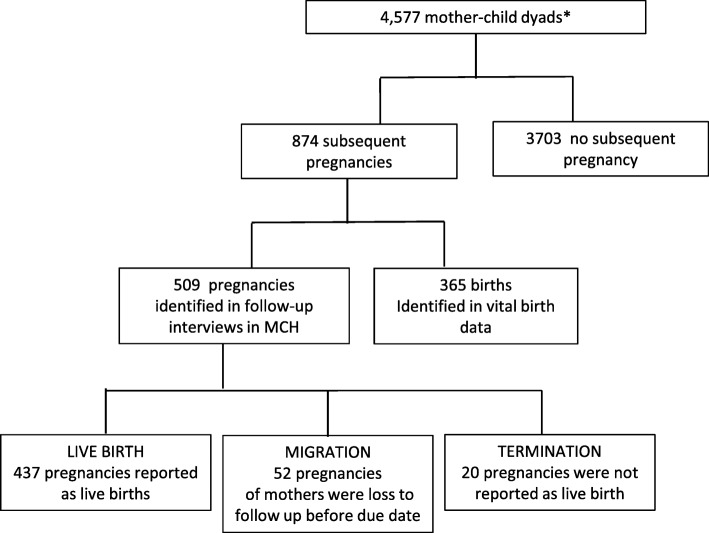
Flowchart of participants. *3719 mothers formed 1 dyad, 405 formed 2 dyads and 16 formed 3 dyads

A total of 4140 NUHDSS female residents aged 15–49 at baseline and their 4577 children born between September 2006 and October 2010 were identified as eligible for enrolment. Among 4140 women, 3719 women had one index child, contributing a single mother-child dyad each, 405 women formed two mother-child dyads and 16 women contributed three dyads. On average, women contributed 19 person-months of follow-up. As shown in Figs. 1, 874 subsequent pregnancies were identified during the follow-up period. Fifty-eight percent (509 pregnancies) were self-reported pregnancies by the women at follow-up interviews and 42% were identified from the NUHDSS’s vital registration data. Among the self-reported pregnancies, 437 were later reported as live births, 52 were loss to follow-up due to out-migration and 20 pregnancies were not reported as live births in the NUHDSS, indicating that they probably ended in miscarriage, abortion or stillbirth.

A summary of selected baseline background characteristics of the women is presented in Table 1. Over one-half of the women lived in the fourth largest slum in Nairobi, Korogocho, and 47% were members of the youthful and mobile population in Viwandani. About 54% of the women were between the ages of 15 and 24 years, and most women reported being currently married or living with partner (85%). Almost one-half (48%) had completed primary school and 23% had attended secondary school or higher education. Concerning ethnicity, Kikuyu was the dominant group, followed by Kamba, Luo and Luhya. One-third of the women had one living child, one-quarter had two and 35% had more than two children. In two-thirds of cases, the index child was aged less than six months at the time of the baseline interview.

**Table 1:** Baseline background characteristics of women of children born between September 2006 and October 2010

	Percent	Number of mother-child dyads
Site		
Korogocho	52.7	2413
Viwandani	47.3	2164
Age		
15–19	15.2	696
20–24	38.5	1761
25–29	27.1	1240
30–49	19.2	880
Marital status		
Married/Living together	85.2	3900
Previously married	5.6	257
Never married	8.9	408
Don’t know/refusal/missing	0.3	12
Education		
Incomplete primary/no education	28.7	1312
Completed primary	47.5	2172
Secondary+	23.3	1068
Missing	0.5	25
Ethnicity		
Kikuyu	25.3	1159
Luhya	17.4	797
Luo	19.8	905
Kamba	21.0	962
Other	16.5	754
Living children		
1	33.2	1521
2	26.7	1220
3+	34.7	1590
Missing	5.4	246
Age of index child		
< 6 months	68.8	3148
6–11 months	26.8	1226
12+ months	4.4	203
Total	100	4577

### Future fertility preferences

Table 2 shows distributions of future reproductive preferences at baseline. One-third of the women wanted to limit reproduction, a similar proportion to that found in the 2012 NCSS. A large majority of women wanted to delay the next child, which is unsurprising in view of the fact that they all had a recent birth. Very few wanted a child soon (2%), one-fifth of the women wanted to wait for two to four years, and more than one-third wanted to wait for at least five years. A small fraction of the women was undecided about future childbearing. About 4% of the women wanted another child but were unsure about the timing (1.1%), or wanted to postpone the next birth until after marriage (2.4%).

**Table 2:** Distributions of future fertility desires

	Percent	Number of mother-child dyads
Future fertility desire		
Want no more	33.3	1526
Undecided	2.8	128
Want another, but timing not specified	1.1	49
Want in 5+ years	37.3	1708
Want in 2–4 years	20.9	958
Want in < 2 years	1.7	78
After marriage	2.4	109
Missing	0.5	21
Total	100.0	4577

There was a clear gradient in perceived severity of an early pregnancy by fertility preference (Table 3). While a small proportion of women wanting no more children said it would be no problem if they got pregnant in the next few weeks (7%), the proportion was 9% among women wanting to delay childbearing for five or more years, 17% among those wanting to wait for two to four years, and 49% among those who wanted another child within two years. Ambivalence was clearer among women who were undecided or wanted to have another but were unsure about the timing: 18 and 29% said it would not be a problem, respectively.

**Table 3:** Distributions of women by how they would feel if they became pregnant in the next few weeks

	Want no more	Undecided	Want another, timing unspecified	Want in 5+ years	Want in 2–4 years	Want in < 2 years	After marriage	Missing	Total	Number of mothers
Big problem	79.2	60.9	55.1	76.7	69.0	39.7	70.6	28.6	74.3	3401
Small problem	3.1	4.7	8.2	5.0	9.9	11.5	0.0	0.0	5.4	246
No problem	6.5	18.0	28.6	9.0	16.7	48.7	3.7	0.0	10.7	491
Can’t get pregnant/no sex	9.8	14.1	6.1	7.4	3.9	0.0	21.1	0.0	7.8	357
Refusal/don’t know	0.1	0.0	0.0	0.0	0.0	0.0	0.0	0.0	0.0	1
Missing	1.4	2.3	2.0	1.8	0.5	0.0	4.6	71.4	1.8	81
Total	100.0	100.0	100.0	100.0	100.0	100.0	100.0	100.0	100.0	4577

In addition, women were asked the main reason why they wanted to space or limit childbearing. Many women reported that the main reason was because they already had too many or enough children, or their babies are too small, while financial reasons were the second most commonly mentioned reason for wanting to limit childbearing (31%) (Table 4). The proportion giving a financial reason was much higher among those wanting no more children or undecided about future childbearing than among those wishing to delay the next birth.

**Table 4:** Reasons for stop or spacing childbearing, according to fertility desire

	Want no more	Undecided	Want another, timing unspecified	Want in 5+ years	Want in 2–4 years	Want in < 2 years	After marriage	Missing	Total	Number of mothers
Financial	30.7	24.6	14.3	12.9	3.8	8.3	11.1	0.0	17.1	778
Related to number or age of existing children*	45.5	47.6	49.0	75.0	90.5	70.8	5.5	0.0	65.2	140
Own health	3.8	2.4	6.1	2.8	2.4	12.5	0.0	0.0	3.1	2967
Infrequent/no sex	1.8	1.6	2.0	0.5	0.5	0.0	8.3	0.0	1.2	53
Other	3.0	6.4	2.0	4.4	1.0	0.0	59.6	0.0	4.5	204
Missing	15.3	17.4	26.5	4.4	1.8	8.3	15.6	100.0	8.9	403
Total	100.0	100.0	100.0	100.0	100.0	100.0	100.0	100.0	100.0	4545

*This category includes responses too many or enough children for limiters, “baby too young” and similar response for spacers

Note: 32 women who wanting a child soon were excluded

### Fertility desires and reproductive outcomes

**Fig. 2:**
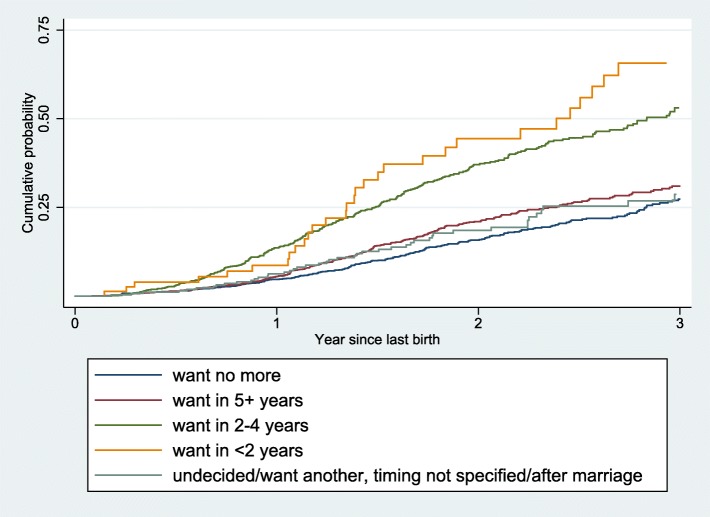
Cumulative probability of pregnancy since last birth by baseline prospective fertility desire

Figure 2 and Table 5 show marked differences in cumulative probability of pregnancy over the three-year period since the index birth by baseline prospective fertility desire. Forty-two percent of women who wanted to have a child within two years and 37% of those wanting in two to four years became pregnant within two years. In contrast, 21% of women wanting to wait for at least five years and 16% of those wanting no more children conceived during the same period. The differences widened steadily over time. For instance, 28% of women wanting no more children and 32% of women wanting to wait for five years or longer became pregnant by the end of three years.

**Table 5:** Cumulative probability of pregnancy since birth of index child by baseline prospective desire

Year since last birth	Want no more	Undecided	Want another, but timing not specified	Want in 5+ years	Want in 2–4 years	Want in < 2 years	After marriage	Total
0	0	0	0	0	0	0	0	0
1	0.05	0.08	0.12	0.05	0.14	0.07	0.01	0.07
2	0.16	0.23	0.29	0.21	0.37	0.42	0.10	0.23
3	0.28	0.39	0.43	0.32	0.56	0.65	0.16	0.37

The cumulative probability of pregnancy within two years was 23% among those who were undecided on having another child, similar to that among women wanting to delay childbearing for five or more years. A high proportion of women who wanted another child but were uncertain about the timing became pregnant within two years (29%). Only 10% of women who wanted to wait until marriage became pregnant. Although there were wide variations between these latter groups, they were combined in Fig. 2 because of the small number of cases.

We performed bivariable and multivariable Cox proportional hazards model analysis to examine the effect of prospective fertility desires on the probability of pregnancy (Table 6). The bivariable analysis showed that prospective desires, site, woman’s age, marital status, woman’s education, ethnicity and number of living children were strongly associated with the probability of pregnancy. Compared with women wanting to delay childbearing for two to four years, women wishing to limit or wanting to delay for at least five years had much lower probability of getting pregnant (0.37 hazard ratio for limiters and 0.48 for the spacers). Living in the mobile settlement Viwandani, having a secondary or higher education, 25 years or older, not currently married, and identifying as Kikuyu ethnicity had significant protective effects from pregnancy compared with the reference groups.

**Table 6:** Hazard ratio of pregnancy since last birth: bivariable and multivariable analysis

	bivariable analysis				Multivariable analysis			
	Hazard ratio	*p*-value	95% CI		Hazard ratio	p-value	95% CI	
Fertility desire								
Want no more	0.37	< 0.001	0.309	0.451	0.50	< 0.001	0.401	0.628
Want in 5+ years	0.48	< 0.001	0.406	0.574	0.57	< 0.001	0.472	0.678
Want in 2–4 years	1.00				1.00			
Want in < 2 years	1.14	0.528	0.757	1.721	1.09	0.688	0.720	1.646
Undecided/want another, timing not specified/after marriage	0.44	< 0.001	0.319	0.621	0.66	0.025	0.466	0.949
Site								
Korogocho	1.00				1.00			
Viwandani	0.64	< 0.001	0.553	0.741	0.66	< 0.001	0.555	0.780
Education								
Incomplete primary/no education	1.00				1.00			
Completed primary	0.86	0.06	0.735	1.007	0.95	0.57	0.801	1.130
Secondary+	0.55	< 0.001	0.446	0.680	0.68	0.001	0.538	0.851
Age								
15–19	1.00				1.00			
20–24	0.89	0.276	0.730	1.094	0.90	0.327	0.718	1.117
25–29	0.71	0.003	0.572	0.891	0.76	0.047	0.584	0.997
30–49	0.60	< 0.001	0.473	0.771	0.62	0.003	0.453	0.845
Living children								
1	1.00				1.00			
2	0.80	0.015	0.667	0.958	0.84	0.077	0.684	1.020
3+	0.85	0.047	0.718	0.998	0.97	0.805	0.762	1.235
Marital status								
Married/Living together	1.00				1.00			
Previously married	0.46	< 0.001	0.313	0.685	0.55	0.004	0.369	0.824
Never married	0.46	< 0.001	0.331	0.630	0.42	< 0.001	0.293	0.597
Ethnicity								
Kikuyu	1.00				1.00			
Luhya	1.81	< 0.001	1.443	2.281	1.60	< 0.001	1.269	2.026
Luo	1.80	< 0.001	1.442	2.256	1.47	0.001	1.165	1.851
Kamba	1.30	0.027	1.030	1.649	1.45	0.004	1.127	1.855
Other	1.99	< 0.001	1.585	2.486	1.46	0.002	1.145	1.867

*N* = 4171

The multivariable analysis revealed that fertility desire had an independent effect on probability of pregnancy. Women wanting no more children and those wanting to wait for five years or longer had 50 and 43%, respectively, lower hazards of becoming pregnant than women wanting to wait for two to four years. The hazard ratio for women who were undecided about future childbearing, wanting another child but being uncertain about the timing, and wanting to wait until marriage was 0.66. There was no significant difference in probability of getting pregnant between women who wanted to wait for two to four years and women wanting to have a child within two years. Place of residence had a significant effect: women living in Viwandani had 34% lower hazard of becoming pregnant compared to women living in Korogocho. Women with secondary or higher education had 32% lower hazard of getting pregnant compared with women with no or incomplete primary education. Unmarried women and women between the ages of 30 and 49 years had lower hazards of pregnancy compared with their respective reference groups. Significant differences were observed by ethnic group: compared with Kikuyu women, women from all other ethnic groups had much higher hazards of getting pregnant. Addition of either strength of desire (whether it would be a big problem if they became pregnant in the next few weeks) or financial concerns as the main reason for spacing or limiting did not improve the fit of the models according to likelihood-ratio tests (results not shown).

Avoidance of pregnancy is of particular importance to women who want no more children or wish to postpone the next birth for a long time. Accordingly, Table 7 focuses on married women wanting no more children or wanting to wait for five years or more before having another child. The hazard ratios were much lower for women with secondary or higher education, compared with their counterparts with no or incomplete primary education (Hazard ratios: 0.63 [95%CI: 0.465–0.842]). Similar to the previous results, being a resident of Viwandani and unmarried, and Kikuyu ethnicity reduced the hazards of pregnancy. There was little difference between those who wanted no more children and those wanting to wait for five or more years in this model.

**Table 7:** Hazard ratio of pregnancy among women wanting to limit/space for 5 years or longer

	Hazard ratio	p-value	95% CI	
Fertility desire				
Want no more	1.00			
Want in 5+ years	1.15	0.234	0.911	1.462
Site				
Korogocho	1.00			
Viwandani	0.66	< 0.001	0.531	0.830
Education				
Incomplete primary/no education	1.00			
Completed primary	0.92	0.481	0.739	1.153
Secondary+	0.63	0.002	0.465	0.842
Age				
15–19	1.00			
20–24	1.13	0.455	0.818	1.564
25–29	0.77	0.186	0.522	1.135
30–49	0.76	0.193	0.495	1.152
Living children				
1	1.00			
2	0.83	0.187	0.623	1.097
3+	1.00	0.994	0.712	1.400
Marital status				
Married/Living together	1.00			
Previously married	0.57	0.011	0.366	0.878
Never married	0.55	0.004	0.366	0.832
Ethnicity				
Kikuyu	1.00			
Luhya	1.53	0.005	1.136	2.054
Luo	1.74	< 0.001	1.314	2.303
Kamba	1.56	0.004	1.157	2.108
Other	1.47	0.025	1.051	2.068

*N* = 2983

## Discussion

Few studies have used a prospective design to improve our understanding of fertility desires among women in sub-Saharan Africa and none has been conducted in a slum population in this region. In this study, we were specifically interested in the extent to which baseline childbearing desires predict subsequent reproductive outcomes. This study makes a significant contribution to assessing the validity of responses to simple questions on future childbearing wishes.

The single most important finding is the strong link between baseline fertility desires and subsequent reproduction. A large difference in pregnancy risk was observed between those who wanted no more children and those who wanted another child. This is in line with previous longitudinal studies conducted in rural Africa: three prospective studies conducted in Nigeria, Ghana and northern Malawi found that preferences, particularly desire to limit childbearing, among married women have strong power to predict childbearing over two to three years period [14, 16, 17]. Women who wanted no more children had one-half or lower odds of getting pregnant in two to three years compared with women wanting more children. Similarly, a study in selected cities in Kenya, Nigeria and Senegal found very large differences in pregnancy risk between women wishing to limit family size and those wanting more children [15]. The single partial exception is a study in rural Mozambique where 56% of women stating at baseline that they wanted no more children gave birth in the three year follow-up compared with 69% of those wanting more [28].

Prior studies in Taiwan, US, South Korea and Sri Lanka between the 1970s and 1990s also showed strong predictive validity of the prospective measure on reproductive outcomes over three to five years [29–33]. More recent similar results have been reported in Egypt and Pakistan [34, 35]. An important conclusion can be reached: the link between woman’s stated desire to stop childbearing and subsequent childbearing is just as strong in sub-Saharan Africa as in other regions of the world.

As well as consolidating the African evidence on the predictive strength of the preference for stopping childbearing, this study advances understanding of the relative effects of desired limitation and spacing or postponement. Nearly all other studies have employed a simple dichotomy of wanting no more children versus wanting more or divided the latter group into those wanting the next child within two years or after two years. Our more detailed classification revealed a pronounced gradient in pregnancy risk according to preferred spacing length. Indeed, the difference in pregnancy risk over three years between women wanting no more children and those wishing to wait for five or more years was marginal: 28% versus 32%.

The importance of this result is underscored by the large proportion of women in the study expressing a desire to wait many years before the next child. This desire was also mentioned by the majority of respondents in a qualitative study in the same slums [11]. In this study, women explained that they would not want to have two children in secondary school at the same time. It may also be true that women want older children to have a degree of independence before another child is born because of the greater challenges in arranging child-care and bringing up children in slums than it is in the rural areas. This result also supports other evidence that the fertility decline in Africa is being driven to an appreciable extent by prolonged spacing or postponement, in contrast to Asia and Latin America where the transition to lower fertility initially took the form of limiting family sizes [36].

Yet implementation of baseline preferences is far from perfect. Among women stating a desire to have no more children, 16% became pregnant within two years, rising to 28% at three years. The corresponding estimates for women wanting to wait for five or more years were 21 and 32%. This finding is consistent with studies in Egypt, Malawi, and Pakistan where between 29 and 34% of women who wanted no more children became pregnant within three years [34, 35, 37]. One possible reason, already mentioned, is that women did genuinely change their minds. However, it is also likely that many women who stated this desire to stop childbearing faced barriers to contraceptive adoption and sustained use. Indeed, another analysis using the same source of data showed a high discontinuation of contraception among postpartum women [38]. It is estimated that unmet need for contraception is about 24% among women in Nairobi’s slums [10]. It is important to note that failure to implement preferences seemed to work in two opposite directions. Many women who at baseline indicated that they wanted a child in less than two years had not gotten pregnant even after three years. Failure to conceive as quickly as desired may be partially responsible; at recruitment 35% of the women were still amenorrheic and for some of these postpartum insusceptibility may have been prolonged. But this result also suggests that prospective preferences change overtime. Our study collected relevant data only at recruitment and cannot assess stability of fertility desire. Additionally, life in the Nairobi slums is unpredictable, incomes fluctuate and relationships are often fragile. It is thus to be expected that changes in circumstances give rise to change in reproductive desires.

The lack of intensity in the desire to protect against future unwanted pregnancy among our study population in the slums is worth highlighting. For example, among postpartum women who wanted to delay child bearing for two to four years about 17% said having a child in the next few weeks would not be a problem, while another 10% said it would only be a small problem. This indicates a tolerance concerning the timing of future childbearing which is consistent with results from other countries including studies among Kenyan women [15]. In a study of three African countries by Speizer [3], Kenyan women reported the highest levels of ambivalence toward childbearing, though the level of contraceptive use was the highest. As suggested by several studies, women with ambivalence are less likely to use contraception or are more likely to discontinue, especially if they face any method-related dissatisfaction [3].

There are very large differences in implementation of preferences by education, locality and ethnicity. We attempted to account for these by examining strength of fertility desires (i.e. big problem or not) but no variation was found. It is not so surprising that educated women had much lower hazard of getting pregnant than the less educated because educated women probably have better knowledge and more agency than less educated women. Other studies among the same slum population showed that women with more education were significantly more likely than others to have adopted a modern contraceptive method, even during the 12 months postpartum [7, 38]. It is possible that, for women with more education, the potential loss of income which will be incurred by time away from the labour force to raise an additional child may be the impetus pushing them to realize their fertility desires [39]. This is supported by the fact that financial reasons were cited as a main reason for limiting by women who wanted no more children. It is, however, worth noting that our results are inconsistent with findings from northern Malawi, where, though contraceptive use was higher among the more educated [40], education did not modify the relationship between reproductive desire and future childbearing [14].

Kikuyu women who wanted to limit family size or postpone the next child for five or more years were significantly more likely to avoid pregnancy than corresponding women from other ethnic backgrounds. This result is consistent with large differentials in contraceptive prevalence between the Kikuyu dominated Central province and the Luo/Luhya dominated Western region but it nevertheless intriguing that similar differences persist when locality and presumably service access is controlled. This suggests cultural factors not yet understood are playing a significant role. Finally, the Viwandani-Korogocho difference is equally intriguing. Even when education, age and ethnicity are controlled for, a big difference persists between the two sites, with women in Viwandani more likely to realise their fertility desires than women in Korogocho. This difference may be partly explained by the lower level of under-five mortality in Viwandani than in Korogocho [22]. Further analysis is therefore needed to understand the specific role community factors play in women (and couples) realising their fertility desires.

This study should be interpreted in light of several limitations. First, the study suffered high loss to follow-up and it is possible that mobile women differ from those who remained in the same slum locations over the observation period. Second, the average follow-up time period was 19 person-months. Although we used survival analysis, the high attrition and short follow-up period prevented the estimation of long-term impact of preferences on reproductive outcomes. Third, we were unable to assess the influence of partners’ preferences on reproductive decisions and outcomes because the interviews were conducted among women. Other studies show that partners’ approval influences woman’s decision to use contraceptive methods and contributes to achieving their fertility desire [41–43]. However, there is also evidence that the influence of a woman and her partner was equal, and in some studies the wife’s preference was found to exert a greater influence than that of her partner [44, 45]. For example, the cross-sectional analysis from 18 national surveys reported few differences in family planning use between couples in whom the wife, but not the husband, wanted more children and vice versa [46]. Fourth, we did not attempt to assess the stability of desires nor the role of contraceptive adoption, failure and discontinuation or abortion in mediating the effect of preferences on reproductive outcomes. Fifth, the eligibility criterion of this study was having a child recently. Such women may be particularly likely to report not wanting to have another child or wishing to wait longer before the next child. Women tend to revise their fertility preferences after they have a new pregnancy [4, 5]. Lastly, the HIV status at the interview was not collected in this nested study. In 2008/2009 HIV prevalence was 12% in the adult population in the two slums [47]. HIV status may have impacted women’s fertility desires and intentions and the implementation of their desire [48, 49].

## Conclusions

Our study adds to the small body of evidence that the stated wishes of women to limit or postpone future childbearing in sub-Saharan Africa have the same meaning and similar predictive validity as preferences in other regions. Concerns that these wishes need not be taken seriously because they are superficial and highly volatile are misplaced. It follows that estimates of unmet need for contraception in Africa are broadly valid and that the increase in the proportion of women in many East African countries who state a desire to have no more children is a trend of considerable importance. In addition, the findings revealed a pronounced gradient in pregnancy risk according to preferred spacing length, which support other evidence on the important contribution of long-term spacing or postponement to fertility decline in sub-Saharan Africa.

## Abbreviations

APHRC: African Population and Health Research Center
DHS: Demographic Health Survey
MCH: Maternal and Child Health
NCSS: Nairobi Cross-sectional Slums Survey
NUHDSS: Nairobi Urban Health Demographic Surveillance System
